# Hematological Indices Predicting the Severity of Acute Pancreatitis Presenting to the Emergency Department: A Retrospective Analysis

**DOI:** 10.7759/cureus.16752

**Published:** 2021-07-30

**Authors:** Noman A Khan, Syed Jawad Haider Kazmi, Muhammad Sohaib Asghar, Manjeet Singh, Shahid Iqbal, Rumael Jawed, Lal Muhammad, Tooba Ahmed Kirmani, Salman Ahmed Khan, Iqra Anees Rajput

**Affiliations:** 1 General Surgery, Liaquat National Hospital and Medical College, Karachi, PAK; 2 Emergency Medicine, Liaquat National Hospital and Medical College, Karachi, PAK; 3 Internal Medicine, Dow University of Health Sciences, Karachi, PAK; 4 Internal Medicine, Liaquat National Hospital and Medical College, Karachi, PAK; 5 Forensic Medicine, Bacha Khan Medical College, Mardan, PAK; 6 Internal Medicine, Dow International Medical College, Karachi, PAK; 7 General Surgery, Civil Hospital Karachi, Karachi, PAK

**Keywords:** pancreas, pancreatitis, severity scores, ct abdomen, hematological indices

## Abstract

Introduction

Acute pancreatitis is defined as inflammation of the pancreas. The body responds to inflammation by producing excessive neutrophils and causing programmed cell death of lymphocytes. This leads to immunological instability, which increases the severity of the disease and mortality rate. Recent data suggest that markers of systemic inflammation are able to predict the prognosis of various diseases. Our study aims to assess the severity of acute pancreatitis in conjunction with these hematological markers of systemic inflammation.

Materials and methods

Our study was carried out in the emergency medicine department of a tertiary care hospital among patients diagnosed with acute pancreatitis. It was a retrospective study done by reviewing the hospital's medical records. Hematological indices such as hemoglobin levels, packed cell volume (PCV), red blood cell (RBC) count, mean corpuscular volume (MCV), mean corpuscular hemoglobin (MCH), mean corpuscular hemoglobin concentration (MCHC), total leukocyte count (TLC), neutrophil count, lymphocyte count, monocyte count, platelet count, neutrophil to lymphocyte count ratio (NLR), lymphocyte to monocyte ratio (LMR), and platelet to lymphocyte ratio (PLR) were observed to be associated with severity of pancreatitis. Those with computed tomography (CT) severity score >=7 were termed as severe pancreatitis, while those below 7 were considered mild to moderate.

Results

A total of 154 patients were included in the final analysis. The mean age of those patients was 48.47 ± 16.71 years. There were 94 male and 60 female patients. There was no difference found among the study groups with respect to mean hemoglobin levels, RBC count, PCV, MCV, MCH, MCHC, lymphocytes, and platelet counts. TLC (p<0.001), neutrophils (p<0.001), monocytes (p=0.008), NLR (p<0.001), and PLR (p=0.006) were found higher in severe pancreatitis, while LMR was found lower in severe pancreatitis (p=0.003). A linear relationship between the hematological indices and CT severity score has shown that TLC (p=0.015), neutrophils (p=0.005), NLR (p=0.001), and PLR (p<0.001) were positively correlated with severity while lymphocyte count (p=0.004) and LMR (p=0.005) were negatively correlated with severe pancreatitis. TLC and LMR were independent predictors of severe pancreatitis with an adjusted odds ratio of 12.80 and 5.47, respectively, on multivariable regression analysis.

Conclusion

Many markers correlated with the CT severity score, but few of them were able to demonstrate statistical significance on receiver operating characteristic (ROC) analysis.

## Introduction

Acute pancreatitis is defined as inflammation of the pancreas ranging from mild degree illness to the severe disease process [1,2]. As acute pancreatitis has a tendency to progress rapidly, there is an urgent need to anticipate severity soon after diagnosis [1-3]. This would result in timely and adequate treatment [1], as well as decreased rates of morbidity and mortality [2]. Not to mention the current overall mortality rate of acute pancreatitis is 2.1% increasing to 17% in severe acute pancreatitis [2,4].

In the past, various scoring systems have been suggested to assess the disease severity. Ranson’s criteria being earliest followed by the Acute Physiology and Chronic Health Evaluation (APACHE) II score, the Modified Glasgow Prognostic Score, the bedside index of severity in acute pancreatitis (BISAP), and Balthazar index [1,3]. Importantly, the Balthazar score, also known as computed tomography (CT) severity score, addresses ongoing necrosis, fluid collections, and inflammation [5]. For the same purpose, many biochemical markers have also been assessed individually, such as C-reactive protein (CRP) and procalcitonin [1]. But mostly, these scoring systems require 48 hours to apply, and their complexity further results in limited use [3,4,6].

The body responds to inflammation by producing excessive neutrophils and causing programmed cell death of lymphocytes. This leads to immunological instability, which increases the severity of disease and mortality rate [7,8]. Recent data suggest that markers of systemic inflammation: neutrophil/lymphocyte ratio (NLR) and platelet/lymphocyte ratio (PLR) are able to predict the prognosis of various diseases, including cancers [1,4,7,9]. In intensive care unit patients, it has been seen that high levels of NLR point towards poor prognosis and high mortality [7,10]. In contrast, PLR predicts prognosis in rheumatic disorders, autoimmune diseases which cause organ damage (e.g., systemic lupus erythematosus (SLE)), multiple infections, and malignancies [7]. In addition, lymphocyte ratio to monocyte (LMR) has also been linked with poor prognosis and increased mortality in patients with cardiovascular diseases, infections like tuberculosis, autoimmune diseases, as well as various malignancies, and more recently in COVID-19 [7]. The levels of these hematological markers could be checked easily with the help of laboratory tests; less time-consuming and affordable [1].

Our study aims to assess the severity of acute pancreatitis in conjunction with these hematological markers of systemic inflammation (NLR, PLR, LMR) in the shortest possible time frame. In the future, this may help clinicians to come up with better management plans for patients with acute pancreatitis. Thus, decreasing the morbidity and mortality in acute pancreatitis.

## Materials and methods

This study was carried out in the emergency medicine department of Liaquat National Hospital among patients diagnosed with acute pancreatitis after admission to the emergency department. It is a retrospective cohort study in a single-center performed by reviewing the hospital's electronic medical records. Patients meeting the clinical criteria of acute pancreatitis whose amylase and lipase values were elevated and radiological diagnoses compatible with acute pancreatitis were included. Demographic data like age and gender were taken from medical records while hematological indices such as hemoglobin levels, packed cell volume (PCV), red blood cell (RBC) count, mean corpuscular volume (MCV), mean corpuscular hemoglobin (MCH), mean corpuscular hemoglobin concentration (MCHC), total leukocyte count (TLC), neutrophil count, lymphocyte count, monocyte count, and platelet count were observed on day 1 of admission. From these values, three ratios were measured such as neutrophil to lymphocyte count ratio (NLR), lymphocyte to monocyte ratio (LMR), and platelet to lymphocyte ratio (PLR). The rest of the clinical data was not part of the analysis. Two study groups were identified on the basis of radiological diagnosis and grading of severity of pancreatitis; those with CT severity score >=7 were termed as severe pancreatitis, while those with below 7 were considered mild to moderate. Those patients whom we did not find sufficient laboratory data or radiological diagnosis, who left the hospital against medical advice, and patients who were under the age of 18 years were excluded from the final analysis.

The statistical analysis was performed with Statistical Package for Social Sciences (SPSS) version 25.0 (IBM Inc., Armonk, New York). The data were checked for normality via the Shapiro-Wilk test. To compare between the study groups, an independent sample t-test was used for quantitative variables. The Chi-square test was used to compare categorical variables. A linear relationship between the parameters and CT severity score was measured via Pearson’s correlation and multiple linear regression. Receiver operating characteristic (ROC) curves were generated to evaluate the diagnostic performance of complete blood picture parameters in determining the severity of pancreatitis. An optimum cut-off value was obtained for significant variables against the area under the curve (AUC). The significant variables were then tested for independent association via univariate and multivariate regression analysis, which reported the odds ratios (OD) and adjusted odds ratios (aOR), respectively, along with their 95% confidence interval (CI).

## Results

A total of 154 patients were included in the final analysis. The mean age of those patients was 48.47 ± 16.71 years. There were 94 male and 60 female patients. There was no difference found among the study groups with respect to mean hemoglobin levels, RBC count, PCV, MCV, MCH, MCHC, lymphocytes, and platelet counts. TLC (p<0.001), neutrophils (p<0.001), monocytes (p=0.008), NLR (p<0.001) and PLR (p=0.006) were found higher in severe pancreatitis, while LMR was found lower in severe pancreatitis (p=0.003) as shown in Table 1.

**Table 1:** Descriptive characteristics of the study population (n=154) Data presented as either mean and standard deviation, or frequency and percentage. M: males; F: females; RBC: red blood cell; PCV: packed cell volume; MCV: mean corpuscular volume; MCH: mean corpuscular hemoglobin; MCHC: mean corpuscular hemoglobin concentration; TLC: total leucocyte count; NLR: neutrophil to lymphocyte ratio; LMR: lymphocyte to monocyte ratio; PLR: platelet to lymphocyte ratio; CT: computed tomography.

Variables	All patients (n=154)	Mild to moderate pancreatitis (n=87)	Severe pancreatitis (n=67)	p-value
Age	48.47 ± 16.71	48.49 ± 17.02	48.43 ± 16.41	0.982*
Gender	M: 94 (61.0%); F: 60 (39.0%)	M: 50 (53.2%); F: 37 (61.7%)	M: 44 (46.8%); F: 23 (38.3%)	0.301^^^
Hemoglobin (g/dL)	12.82 ± 2.78	12.62 ± 2.35	13.08 ± 3.27	0.340*
RBC count (_x_10^^6 ^cells/uL)	4.67 ± 1.01	4.54 ± 0.79	4.84 ± 1.22	0.092*
PCV (%)	39.50 ± 8.26	39.03 ± 7.32	40.11 ± 9.39	0.453*
MCV (fL)	84.92 ± 7.35	85.98 ± 6.14	83.52 ± 8.55	0.056*
MCH (pg)	27.61 ± 2.93	27.88 ± 2.55	27.25 ± 3.35	0.216*
MCHC (g/dL)	32.45 ± 1.69	32.36 ± 1.48	32.58 ± 1.93	0.450*
TLC (_x_10^^3^/uL)	14.65 ± 6.70	12.85 ± 5.94	17.02 ± 6.96	<0.001*
Neutrophils (cells/uL)	12.22 ± 6.36	10.40 ± 5.63	14.61 ± 6.52	<0.001*
Lymphocytes (cells/uL)	1.64 ± 0.85	1.75 ± 0.84	1.49 ± 0.85	0.071*
Monocytes (cells/uL)	0.62 ± 0.30	0.56 ± 0.29	0.70 ± 0.30	0.008*
Platelet count (_x_10^^3^/uL)	264.58 ± 95.06	257.34 ± 95.37	274.12 ± 94.55	0.292*
NLR	10.32 ± 9.78	7.74 ± 7.68	13.72 ± 11.18	<0.001*
LMR	3.24 ± 2.40	3.75 ± 2.38	2.58 ± 2.28	0.003*
PLR	206.32 ± 141.85	176.90 ± 113.59	245.09 ± 165.25	0.006*
CT severity score	6.74 ± 1.78	5.43 ± 0.84	8.44 ± 1.13	<0.001*
CT severity <7	-	87 (56.5%)	-	-
CT severity >=7	-	-	67 (43.5%)	-

A linear relationship between the hematological indices and CT severity score has shown that TLC (p=0.015), neutrophils (p=0.005), NLR (p=0.001), and PLR (p<0.001) were positively correlated with severity while lymphocyte count (p=0.004) and LMR (p=0.005) were negatively correlated with severe pancreatitis. On multiple linear regression, out of them, only TLC, lymphocytes, NLR, and PLR were found associated with CT severity score. Other than them, platelet count and monocytes showed association, as shown in Table 2.

**Table 2:** Correlation and multiple linear regression of hematological indices with a dependent variable of CT severity score * indicates a significant p-value of less than 0.05. ^ indicates p-value calculated by Pearson’s correlation. † indicates p-value calculated by Linear regression model with CT severity score as the dependent variable. R: regression coefficient; B: unstandardized coefficient; β: standardized coefficient; S.E: standard error; RBC: red blood cell; PCV: packed cell volume; MCV: mean corpuscular volume; MCH: mean corpuscular hemoglobin; MCHC: mean corpuscular hemoglobin concentration; TLC: total leucocyte count; NLR: neutrophil to lymphocyte ratio; LMR: lymphocyte to monocyte ratio; PLR: platelet to lymphocyte ratio; CT: computed tomography.

Model	R	p-value^^^	B	S.E	β	t-statistic	p-value^†^	95% confidence interval for B
(Constant)	-	-	4.718	26.288	-	0.859	0.858	-47.287 – 56.723
Hemoglobin	0.020	0.807	1.335	1.047	2.079	1.275	0.204	-0.736 – 3.406
RBC count	0.082	0.322	0.307	1.405	0.174	0.218	0.828	-2.473 – 3.086
PCV	0.008	0.921	-0.478	0.394	-2.210	-1.212	0.228	-1.259 – 0.302
MCV	-0.153	0.065	0.169	0.266	0.695	0.635	0.526	-0.357 – 0.695
MCH	-0.105	0.205	-0.650	0.734	-1.067	-0.885	0.378	-2.103 – 0.803
MCHC	0.040	0.634	0.150	0.756	0.142	0.198	0.843	-1.346 – 1.645
TLC	0.201	0.015*	1.227	0.550	4.602	2.230	0.027*	0.138 – 2.316
Neutrophils	0.233	0.005*	-1.020	0.546	-3.631	-1.869	0.064	-2.100 – 0.060
Lymphocytes	-0.237	0.004*	-1.382	0.642	-0.622	-2.153	0.033*	-2.652 – -0.112
Monocytes	0.090	0.280	-2.370	1.170	-0.402	-2.025	0.045*	-4.685 – -0.055
Platelet	0.037	0.660	-0.005	0.002	-0.288	-2.212	0.029*	-0.010 – -0.001
NLR	0.282	0.001*	-0.139	0.052	-0.762	-2.682	0.008*	-0.242 – -0.037
LMR	-0.229	0.005*	-0.025	0.114	-0.033	-0.216	0.829	-0.250 – 0.201
PLR	0.299	<0.001*	0.011	0.003	0.869	3.445	0.001*	0.005 – 0.017

**Figure 1:**
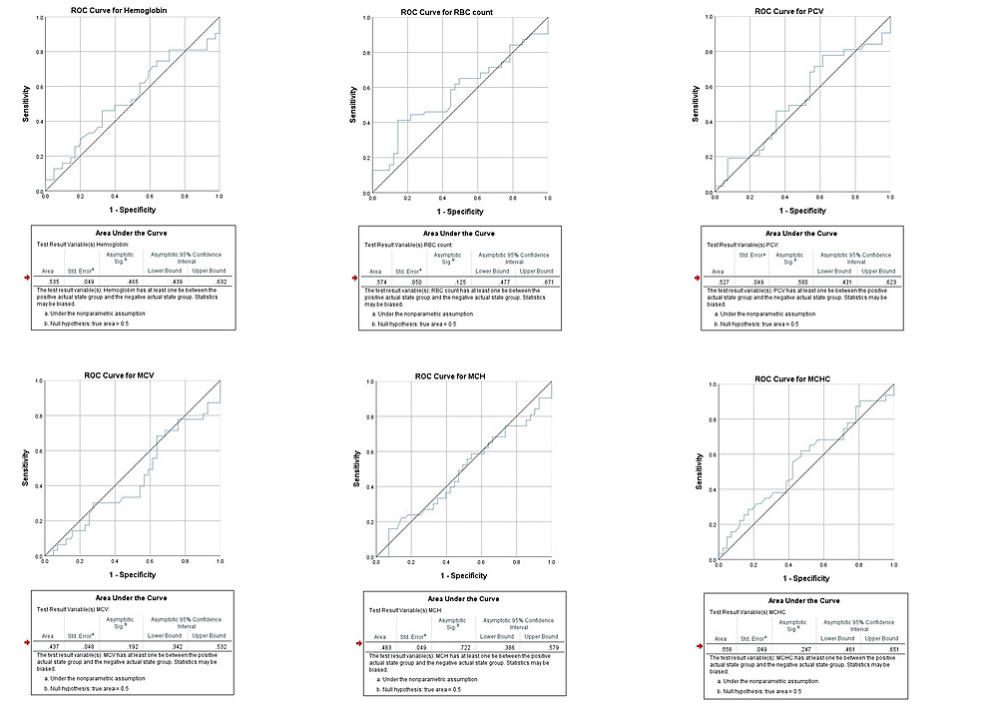
Individual ROC curves of study variables for severe pancreatitis indicating area under the curve, its 95% confidence interval, standard error and statistical significance respectively. ROC: receiver operating characteristic; RBC: red blood cell; PCV: packed cell volume; MCV: mean corpuscular volume; MCH: mean corpuscular hemoglobin; MCHC: mean corpuscular hemoglobin concentration.

**Figure 2:**
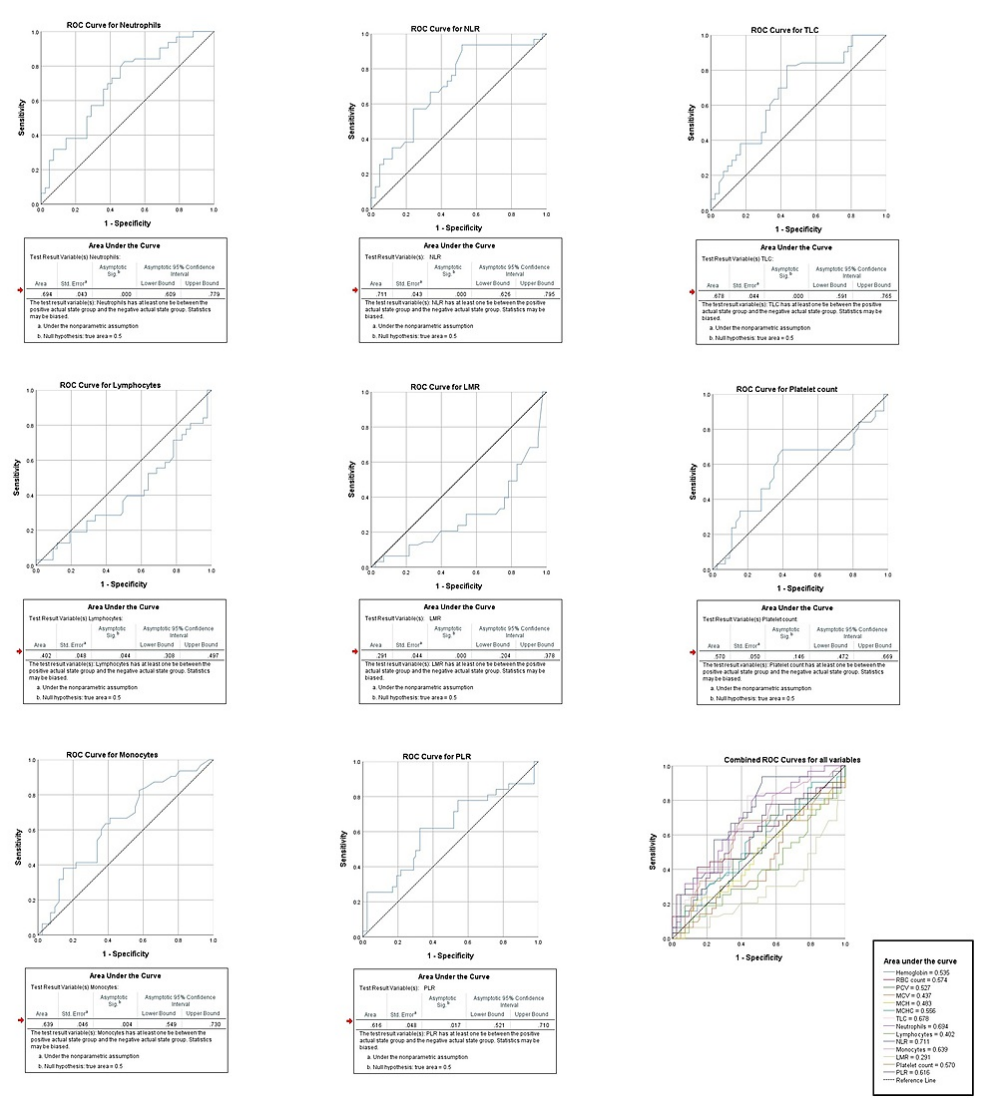
Individual and combined ROC curves of study variables for severe pancreatitis indicating area under the curve, its 95% confidence interval, standard error and statistical significance respectively. ROC: receiver operating characteristic; TLC: total leucocyte count; NLR: neutrophil to lymphocyte ratio; LMR: lymphocyte to monocyte ratio; PLR: platelet to lymphocyte ratio.

Receiver operating characteristic analysis shows highest area under the curve for NLR (0.711), followed by neutrophils (0.694), and TLC (0.678). It was also found significant for monocytes (0.639), PLR (0.616), lymphocytes (0.402), and LMR (0.291), as shown in Figure 1-2.

Further, the optimum cut-off was determined using the ROC curves, and multivariate regression was carried out using significant variables, with findings suggestive of only TLC and LMR been independent predictors of severe pancreatitis with an adjusted odds ratio of 12.80 and 5.47, respectively. The rest of the predictors were significant on univariate regression only when they were not adjusted for age, gender, and each other, as shown in Table 3.

**Table 3:** Multivariate regression of hematological indices for CT severity score >=7 (severe pancreatitis) * Cut-offs determined by Receiver operating curves (only significant variables opted for multivariate analysis). ^ Dependent variable is severe pancreatitis (CT severity score >=7). † Model is adjusted for age, gender, and all variables with each other. OR: odds ratio; aOR: adjusted odds ratio; CI: confidence interval; CT: computed tomography; TLC: total leucocyte count; NLR: neutrophil to lymphocyte ratio; LMR: lymphocyte to monocyte ratio; PLR: platelet to lymphocyte ratio.

Variables	^^^OR	95% CI		p-value	^†^aOR	95% CI		p-value
		Upper	Lower			Upper	Lower	
TLC >11.73*	6.172	2.823	13.491	<0.001	12.807	2.031	80.783	0.007
Neutrophils >10.41*	3.900	1.922	7.914	<0.001	0.285	0.045	1.802	0.182
Lymphocytes <1.66*	2.125	1.091	4.139	0.027	0.831	0.254	0.033*	0.760
Monocytes >0.55*	2.882	1.457	5.704	0.002	0.599	0.197	1.819	0.366
NLR >7.94*	3.929	1.963	7.863	<0.001	0.814	0.249	2.667	0.735
LMR <2.44*	6.300	3.047	13.024	<0.001	5.478	1.627	18.442	0.006
PLR >177.16*	3.370	1.699	6.687	0.001	1.625	0.582	4.536	0.354

## Discussion

The current study focused only on the hematological predictors of acute pancreatitis due to their cost-effectiveness and early recognition of the severity of the disease. Multiple studies conducted in the past were able to associate different hematological markers with the severity of acute pancreatitis. A retrospective analysis was conducted in India with 107 patients linked NLR with severity and mortality in acute pancreatitis [11]. Value of NLR above 8.5 was considered 78% sensitive and 70% specific for severe disease in the same study. Another study conducted on 557 patients concluded both NLR and PLR are associated with severity and mortality with equal sensitivity and specificity [4]. When the same markers were studied in the pregnant population, NLR was significantly elevated in acute pancreatitis, but there was no statistically significant difference in terms of PLR [12]. Further, a sensitivity of 71.4% and specificity of 100.0% was determined at a cut-off NLR value to be 4.10 [12]. Jeon, et al. [13], in their analysis, predicted NLR >4.76 with severe pancreatitis and >4.88 with organ failure.

Recently, the severity of acute pancreatitis is majorly based on the Atalanta classification system, including Ranson’s criteria score >3, APACHE-II score >8, or organ failure and local pancreatic pathology (like an abscess, pseudocyst, necrosis, etc.) [14]. Predictive markers can also distinguish among the etiology of pancreatitis. One such study conducted in the Korean population demonstrated both NLR and PLR were significantly associated with gall stone pancreatitis as opposed to alcohol-induced pancreatitis [15]. Another study from Turkey shown NLR, PLR, and TLC predictive of severity and complications with acute pancreatitis [16]. In the current study, we have found significant associations of TLC and LMR on multivariable regression analysis. One study did not favor the laboratory markers on admission had a prognostic value, rather NLR, PLR, and CRP/albumin ratio at 48 hours of hospital stay were significantly associated with acute pancreatitis complications [3]. On the contrary, one such study did not found any correlation of hematological indices with the severity of acute pancreatitis [17]. Another study on acute pancreatitis in pregnancy has modulated a risk score calculation based on TLC, neutrophils, LMR, and other independent markers associated with severity with an AUC of 0.906, aOR of 3.013, specificity of 82.8%, and sensitivity of 87.5% [18]. In our results, we were able to predict the severity of acute pancreatitis similar to the above-quoted study with TLC having aOR of 12.80 and LMR having aOR of 5.47, respectively. With respect to basic hematological indices, hemoglobin levels and RBC count were not associated with severe pancreatitis according to one study, but TLC and MCV were found statistically different in the severe group [19]. The current study did not find any relation of RBC count, MCV, PCV, MCH, MHCH, and hemoglobin levels in discriminating the severity of pancreatitis.

Our findings of NLR and LMR being associated with severe acute pancreatitis were also supported by Li, et al. [1], where NLR had the largest AUC of 0.804 with a cut-off value of 16.64 having 82.4% sensitivity and 75.6% specificity. With our results, we have also shown the highest AUC for NLR (0.711) at a cut-off value of 7.94. TLC was also significantly associated with severe acute pancreatitis at cut-off 12.10 with AUC of 0.723, sensitivity of 64.7%, specificity of 67.7%, positive predictive value of 52.4%, negative predictive value of 77.8%, and accuracy of 66.7% [20]. This finding was also similar to our results, where TLC at cut-off 11.73 had an AUC of 0.678. Patients with raised NLR not only had an increased risk of mortality but also had increased length of hospital stay in a retrospective audit conducted in Ireland [6]. Both LMR <2.0 and NLR > 10.5 showed a significant difference with complicated acute pancreatitis [21]. Certain studies have identified immature granulocytes have more power to discriminate severity of acute pancreatitis than TLC or NLR [2,22]. Lastly, when compared to the control group, TLC, NLR, and platelet count all shown a significant difference in this study. However, when correlated with severe and non-severe groups of pancreatitis, only NLR and TLC were found significant [23]. In severe acute pancreatitis, the cut-off value of NLR was 8.05 with a sensitivity of 93%, specificity of 86%, and an AUC of 0.937 [23]. Other novel markers that have been studied in the literature but weren't part of our analysis included red cell distribution width (RDW) [6,19], mean platelet volume (MPV) [20], and immature granulocytes [2,22].

The current study had a few limitations, such as a single-center review of cases and a limited sample size. Despite limited generalizability, the study had enough power of statistical significance to predict the outcomes as the study relied solely on the radiological evidence of the severity of the disease and did not rely on the clinical parameters or other score-based severity indexes, which might have less sensitivity. There might be a selection bias inconsequential for our study because we included only those patients that have undergone CT scans for diagnosis. However, this is the first study that studied the whole complete blood picture indices to associate with the severity of the disease, excluding the other non-specific markers of inflammation.

## Conclusions

With the majority of literature was evident of NLR being the major indicator of inflammatory response in severe pancreatitis, our results more independently associated TLC and LMR with severe pancreatitis. Many markers correlated with the CT severity score, but few of them were able to demonstrate statistical significance on ROC analysis. A prospective validation cohort study is, however, required on a large sample population to reproduce these findings as a causal association.
